# The efficacy of colchicine in the management of coronavirus disease 2019: A protocol for systematic review and meta-analysis

**DOI:** 10.1097/MD.0000000000021911

**Published:** 2020-09-04

**Authors:** Mohamed Nabil Elshafei, Ahmed Khalil, Ahmed El-Bardissy, Mohammed Danjuma, Mohamed Badie Ahmed, Mouhand F.H. Mohamed

**Affiliations:** a Clinical Pharmacy Department. Hamad General Hospital; b Internal Medicine Department, Hamad Medical Corporation; c College of Medicine, QU Health, Qatar University, Doha, Qatar.

**Keywords:** colchicine, coronavirus disease 2019, mortality, severe acute respiratory syndrome coronavirus 2

## Abstract

**Background:**

Severe acute respiratory syndrome coronavirus 2 (SARS-COV2) infection is a recently emerged viral infection causing predominantly mild upper respiratory symptoms. However, in some instances, it might result in acute respiratory distress syndrome (ARDS) that poses a significant mortality risk. ARDS is postulated to be mediated by a surge of pro-inflammatory cytokines and chemokines, leading to a dysregulated hyper inflammatory response. Colchicine being an anti-inflammatory agent, might mitigate this dysregulated response. Thus, in the absence of therapeutic options available to manage coronavirus disease 2019 (COVID-19), it is imperative to ascertain the effect of colchicine on improving outcomes in COVID-19 patients.

**Method:**

We will perform a systematic review including a search of the following databases: PubMed, EMBASE, MEDLINE, Clinicaltrials.gov, Cochrane library, and google scholar since inception. We will include randomized controlled trials exploring the effect of colchicine on the efficacy and safety outcomes of COVID-19 patients. Subsequently, we will perform a meta-analysis utilizing the random-effects to ascertain the effect of colchicine on reducing COVID-19 related mortality (primary endpoint) and other efficacy and safety outcomes.

**Results:**

Our review results are anticipated in early 2021 (based on the completion of several ongoing randomized controlled trial). Our review results will be published in a peer-reviewed journal.

**Conclusion:**

This systematic review and meta-analysis, is exploring the effect of colchicine on the efficacy and safety outcomes of COVID-19 patients. If colchicine proved to be effective, it would be a significant milestone in the management of COVID-19, a disease with limited available therapeutic options.

**PROSPERO registration number:**

CRD42020191086

## 1 Introduction

Severe acute respiratory syndrome coronavirus 2 (SARS-CoV-2), a novel coronavirus, is a member of the coronavirus family that infects humans and animals. It is a single-stranded RNA virus that was discovered in Wuhan, China, in December 2019, and now has become a pandemic as declared by the World Health Organization (WHO) on March 11, 2020. As of June 28, 2020, nearly ten million individuals tested positive worldwide, with half a million reported deaths^[1]^

Acute respiratory distress syndrome (ARDS) is a life-threatening acute, diffuse, inflammatory form of lung injury. It is a common cause of mortality in coronavirus disease 2019 (COVID-19) pneumonia patients. ARDS occurs as a result of SARS-COV2 infection-induced acute inflammation in the lung (Goh et al, 2020).^[2,3]^ This inflammation is mediated by pro-inflammatory cytokines such as interleukin 1 (IL1), interleukin 6 (IL6) and tumor necrosis factor (TNF) and could be very deleterious if left unopposed.^[3,4]^ Therefore, early recognition and management are crucial to reduce associated morbidity and mortality.

Thus, it has always been a challenge to identify medication with potent anti-inflammatory properties, which might have beneficial effects in COVID -19 patients. These features have led many researchers to consider the potential role of the colchicine in the management of SARS-CoV-2 infection.^[5–10]^ Colchicine, a lipid-soluble alkaloid, accumulates in granulocytes and monocytes (in different concentrations in comparison with plasma levels) after 24 to 72 hours of oral administration with resulting anti-inflammatory effects. Colchicine has an impact on the chemotaxis of inflammatory cells, such as neutrophils and monocytes. It also blocks E-selectin expression, which is responsible for binding leukocytes to endothelial cells, attracting monocytes and neutrophils to inflamed tissue, and inhibiting the release of IL-1β and IL-18.^[11,12]^ Colchicine use is established in several clinical settings, including gout, rheumatic diseases, and pericarditis.^[13–17]^

Recently, Deftereos et al conducted a small-sized randomized controlled trial (RCT) evaluating the effect of colchicine on cardiac and inflammatory biomarkers and clinical outcomes in patients hospitalized with COVID-19.^[6]^ This small-sized study of 105 patients showed improvement in time to clinical deterioration in patients who received colchicine with no significant differences in the biomarkers’ levels. Moreover, a clinical.trial.gov query reveals that there are more than ten ongoing RCT examining the effect of colchicine in the context of COVID-19. Hence, we aim to perform a systematic review and a meta-analysis to evaluate the efficacy of colchicine in the management of COVID-19.

## 2 Methods

This protocol is adherent to the Preferred Reporting Items for Systematic Reviews and Meta-Analyses guidelines (PRISMA).^[18]^ It is also registered with the International Prospective Register of Systematic Reviews (registration number: CRD42020191086).

## 3 Inclusion criteria

### 3.1 Type of studies

We will limit our review to RCTs comparing colchicine to the standard of care or placebo in adult patients with COVID-19. We will limit our inclusion to articles written in the English language.

### 3.2 Type of participants

Adult patients (>18 years) with Confirmed SARS-CoV-2 infection.

### 3.3 Type of intervention

Colchicine therapy.

### 3.4 Type of comparator

Standard of care or Placebo.

### 3.5 Type of outcome measures

#### 3.5.1 Primary outcome

Risk of mortality

#### 3.5.2 Secondary outcomes

We will look at other outcomes, including the risk of admission to the intensive care unit (ICU), risk of intubation, risk of adverse drug reactions (ADR), rate of remission.

## 4 Exclusion criteria

We will exclude all other studies that fail to meet the inclusion criteria, as mentioned above.

## 5 Information sources and literature search

A comprehensive search of the following databases will be attempted: PubMed, MEDLINE, EMBASE, Clinicaltrials.gov, Cochrane library, and google scholar (first 200 relevant hits). We will use free text, emtree, and MesH terms in our search. There will be no language or date limitations implied in the search. Example of a proposed search strategy is (((COVID-19) OR (SARS-CoV-2)) OR (coronavirus disease 2019)) AND (((colchicine) OR (Colcrys)) OR (Mitigare)). We will also perform relevant citations and reference searches.

## 6 Screening and data extraction

Two reviewers (MNE) and (AHE) will conduct the search in 2 stages. The first stage will be screening the retrieved articles’ titles and abstracts in an independent manner. In the second stage, the articles’ full text will be retrieved and further assessed for eligibility. At any stage, if a disagreement occurs, a third reviewer (AAK) will resolve it guided by the protocol. We will use excel sheets to collect pertinent data. This includes author name, publication date, study location, sample size, events number, demographics (age, gender, weight, and comorbidities), COVID-19 severity, colchicine dosing, outcome definition, comparator's details, and follow-up duration.

## 7 Study quality and risk of bias assessment

We will utilize the Cochrane risk of bias assessment tool to assess the study quality and risk of bias.^[19]^ Moreover, we will grade the quality of evidence using the GRADE approach.^[20]^ Finally, we will generate funnel plots to explore the risk of publication bias.

## 8 Statistical analyses

We will use the risk ratio with 95% confidence interval, to pool the effect of colchicine compared to comparators. We will use the *I*^2^ to examine heterogeneity between studies. *I*^2^ > 60% will suggest a marked heterogeneity. Regardless of the heterogeneity level, we will utilize the random-effects model in our analysis. MetaXl software will be used for statistical analysis (version 5.3 © EpiGear International Pty Ltd ABN 51 134 897 411 Sunrise Beach, Queensland, Australia, 2011–2016).

## 9 Subgroup and sensitivity analyses

We will perform sensitivity analysis to reflect the relative constituent studies’ extent on the overall point estimate of our outcome. Additionally, we will perform a subgroup analysis if data permit (ICU admitted patients, analysis excluding studies with a high risk of bias, gender, age categories).

## 10 Ethics

Formal ethical approval is not needed for our review since it is a synthesis of already available data.

## 11 Discussion

In this review, we will explore the efficacy and safety of colchicine in COVID-19 patients. The mortality is our primary outcome; nonetheless, we will be examining many other outcomes. Colchicine is an efficacious, affordable, available, and safe anti-inflammatory agent. Thus, repurposing this medication to combat the inflammatory response associated with COVID-19 has been of interest to the medical community. This is evident by the number of ongoing RCTs assessing its utility in the management of SARS-CoV-2 infection. If our review revealed a positive effect of colchicine, this would be a big step adding to the scarce COVID-19 available therapeutic modalities.^[21]^ The results of this review and its constituent studies are of more value in limited-resource settings where expensive alternatives (such as Remdesivir) are not available or affordable.^[22,23]^ We anticipate completing the review in early 2021 (depending on the availability of results of ongoing RCTs ). The results of this review will be presented at conferences and will be published in a peer-reviewed journal.

## Acknowledgments

We acknowledge the Qatar National Library (QNL) for funding the open access fees of this publication.

## Author contributions

**Conceptualization:** Mohamed Nabil, Mohammed Danjuma

**Formal analysis:** Mouhand F.H Mohamed

**Methodology:** Mouhand F.H Mohamed, Mohamed Badie

**Project administration:** Ahmed El-Bardissy, Ahmed Khalil

**Review and editing**: Mouhand F.H Mohamed, Mohamed Nabil

**Supervision:** Mouhand F.H Mohamed, Mohammed Danjuma

**Writing – original draft:** Mohamed Nabil, Ahmed El-Bardissy, Ahmed Khalil
